# Integrating dynamic SOFA changes and age to predict 28-day mortality in ICU patients: a nomogram and machine learning validation study

**DOI:** 10.3389/fmed.2025.1707548

**Published:** 2026-01-26

**Authors:** Yu Xu, Man Chen, Kang Xu, Jing Chu, Jianying Guo

**Affiliations:** 1 Department of Pulmonary and Critical Care Medicine, Longgang Central Hospital of Shenzhen, Shenzhen, Guangdong, China; 2 Department of Critical Care Medicine, Hebei Medical University Third Hospital, Shijiazhuang, China

**Keywords:** dynamic, SOFA, segment, nomogram, XGBoost

## Abstract

**Background:**

The Sequential Organ Failure Assessment (SOFA) score is widely used to predict prognosis in critically ill patients, but the prognostic value of dynamic SOFA changes (Δ SOFA) and their integration into prediction models remains unclear.

**Methods:**

This retrospective study included 665 ICU patients admitted to the Third Hospital of Hebei Medical University between July 2022 and December 2023. The initial and daily SOFA scores (days 1–3) and demographic data were collected. Patients were stratified by SOFA 1 scores (4–7, 8–11, ≥12). A nomogram combining SOFA1, ΔSOFA 3–1, and age was developed, and its discriminative ability and calibration were evaluated. Additionally, an XGBoost model using the same predictors was constructed to explore the potential value of machine learning. External validation was performed using the MIMIC-IV database.

**Results:**

Overall, the 28-day mortality rate was 18.9%. Mortality increased with higher SOFA 1 and ΔSOFA 3–1 scores. The nomogram showed high discriminative ability (C-index: 0.852 for SOFA 1 = 4–7; 0.845 for SOFA 1 = 8–11) and good calibration. The optimized XGBoost model exhibited excellent discriminative performance in the internal training cohort, with an area under the curve (AUC) of 0.833. The AUC was 0.863 in the independent internal test cohort and 0.671 in the external validation cohort. SHAP analysis identified ΔSOFA 3–1 as the most influential predictor across the datasets.

**Conclusion:**

Dynamic changes in SOFA scores (ΔSOFA 3–1), especially in patients with moderate baseline SOFA 1 scores (4–11), significantly improve prognostic accuracy when combined with age. The nomogram provides an intuitive bedside tool for early risk stratification, whereas the XGBoost model demonstrates the potential value of machine learning. External validation highlights the need for further multicenter studies to enhance model generalizability.

## 1 Introduction

Patients admitted to the Intensive Care Unit (ICU) often present with life-threatening conditions and a high risk of mortality, highlighting the critical need for accurate outcome prediction tools. Among the most widely used scoring systems is the Acute Physiology and Chronic Health Evaluation II (APACHE II), which estimates mortality based on the most abnormal physiological parameters within the first 24 h of ICU admission (1). However, APACHE II does not account for the evolution of a patient’s condition during ICU stay, thus limiting its utility in dynamic clinical decision-making (2).

The Sequential Organ Failure Assessment (SOFA) score (3), introduced in 1994 through international consensus, was originally intended to monitor changes in organ function over time rather than predict outcomes (4). Over the years, SOFA has become a widely adopted tool in ICUs, with numerous studies demonstrating its association with patient prognosis (5–8). Dynamic tracking of SOFA scores can improve prognostic accuracy (9, 10). However, the relationship between score changes and patient mortality is not linear (11). For example, a declining SOFA score does not always indicate improved outcomes, particularly in patients with extremely high or low initial scores (12). Ferreira et al. (13) found that patients with an initial SOFA score greater than 11 had a mortality rate of 95%, regardless of subsequent changes. Likewise, a recent randomized controlled trial by Liu et al. (14) involving 1,817 sepsis patients with SOFA scores ranging from 2 to 13 showed a significant reduction in 28-day mortality with hemopexin treatment. These studies suggest that the prognostic value of dynamic SOFA changes may depend heavily on the initial score range, warranting further research into SOFA trajectory stages that are most predictive of patient outcomes.

To bridge this knowledge gap, this study aims to evaluate the prognostic relevance of dynamic SOFA score changes among critically ill patients in a comprehensive ICU setting. Specifically, we sought to identify the SOFA score intervals in which dynamic SOFA changes provide the most accurate mortality prediction and to construct a reliable, age-adjusted prognostic model based on dynamic SOFA trends.

## 2 Materials and methods

### 2.1 Study population and data collection

This retrospective observational study was conducted at the Third Hospital of Hebei Medical University, a tertiary teaching hospital with a 29-bed comprehensive intensive care unit (ICU). Patients admitted to the ICU between July 2022 and December 2023 were screened. Patients were excluded if they underwent organ donation or had an ICU stay of less than 3 days.

Demographic data and disease classifications were retrieved from the hospital information system (HIS), along with APACHE II scores recorded within 24 h of ICU admission. Sequential Organ Failure Assessment (SOFA) scores were calculated daily based on clinical and laboratory parameters across six organ systems: central nervous, respiratory, hepatic, renal, coagulation, and cardiovascular systems.

Neurological status was evaluated using the Glasgow Coma Scale (GCS) by attending physicians and nursing staff. The GCS score was documented without sedation or estimated if sedation was present.

Each organ system was scored on a scale of 0 (normal) to 4 (most abnormal), yielding a total SOFA score ranging from 0 to 24. The hospital’s clinical information system (ICCA) automatically computed SOFA scores by extracting hourly data.

SOFA scores were automatically calculated by the system (ICCA) by retrieving data every hour. Initial SOFA scores (scores at admission to the ICU) were generated from data obtained within the first 1–2 h after admission, with missing data treated as a score of 0. Subsequent daily SOFA scores were collected at 07 a.m. on each follow-up day. Prognosis, including both survival and mortality outcomes, was tracked for 28 days post-admission via the hospital information system or telephone follow-ups. The delta SOFA (ΔSOFA) for any follow-up day was calculated using the formula (SOFA score of the follow-up day－day 1 SOFA).

#### 2.1.1 External validation data

External validation was performed using the Medical Information Mart for Intensive Care IV (MIMIC-IV, version 3.1) database. MIMIC-IV is a large, publicly accessible, de-identified critical care database containing comprehensive clinical information of patients admitted to the intensive care units (ICUs) of the Beth Israel Deaconess Medical Center in Boston, Massachusetts, United States, between 2008 and 2022. For external validation, we applied the same inclusion and exclusion criteria as those used for our institutional cohort to identify eligible patients with MIMIC-IV. Access to MIMIC-IV was granted after completion of the required training and data use agreement (certification number: 69733112).

### 2.2 Statistical analysis

Statistical analysis was performed using IBM Statistical Package for the Social Sciences (SPSS) version 27.0 and R statistical software (R Foundation for Statistical Computing, Vienna, Austria). Categorical variables were presented as numbers (percentage, %) and compared using the chi-square test. Normally distributed continuous variables were expressed as mean ± standard deviation (x̄ ± SD) and compared using *t*-tests. The risk of death was evaluated using receiver operating characteristic (ROC) curve analysis. The DeLong test was used to statistically compare the AUCs between the different models. Multifactorial logistic regression analysis was conducted to identify risk factors. Statistical significance was set at a *p*-value of <0.05. Smoothed curve fitting was performed using the EmpowerStats statistical software,^1^ and a nomogram was developed using the “rms” package in R statistical software.

The XGBoost model (15) (binary: logistic objective) was developed in Python 3.13 and integrated within R (version 4.5) via the reticulate package. Data were randomly split into a training set and an internal validation set (70:30 split). Model performance was evaluated by calculating the area under the receiver operating characteristic curve (AUC), both in the internal validation set and in an external validation cohort, to assess the generalizability of the model.

SHapley Additive exPlanations (SHAP) analysis was performed to interpret the contribution of each feature to the model predictions using summary plots and feature importance visualizations.

All modeling, validation, and interpretation were conducted in Python using packages including XGBoost, SHAP, scikit-learn, pandas, and matplotlib.

## 3 Results

During the study period from July 2022 to December 2023, 1,177 patients were admitted to the ICU of the Third Hospital of Hebei Medical University. After excluding 190 patients who underwent organ donation and 322 patients with ICU stays shorter than 3 days, 665 patients were included in the final analysis. Among these, 126 patients (18.9%) died, and 539 (81.1%) survived.

**Figure 1:**
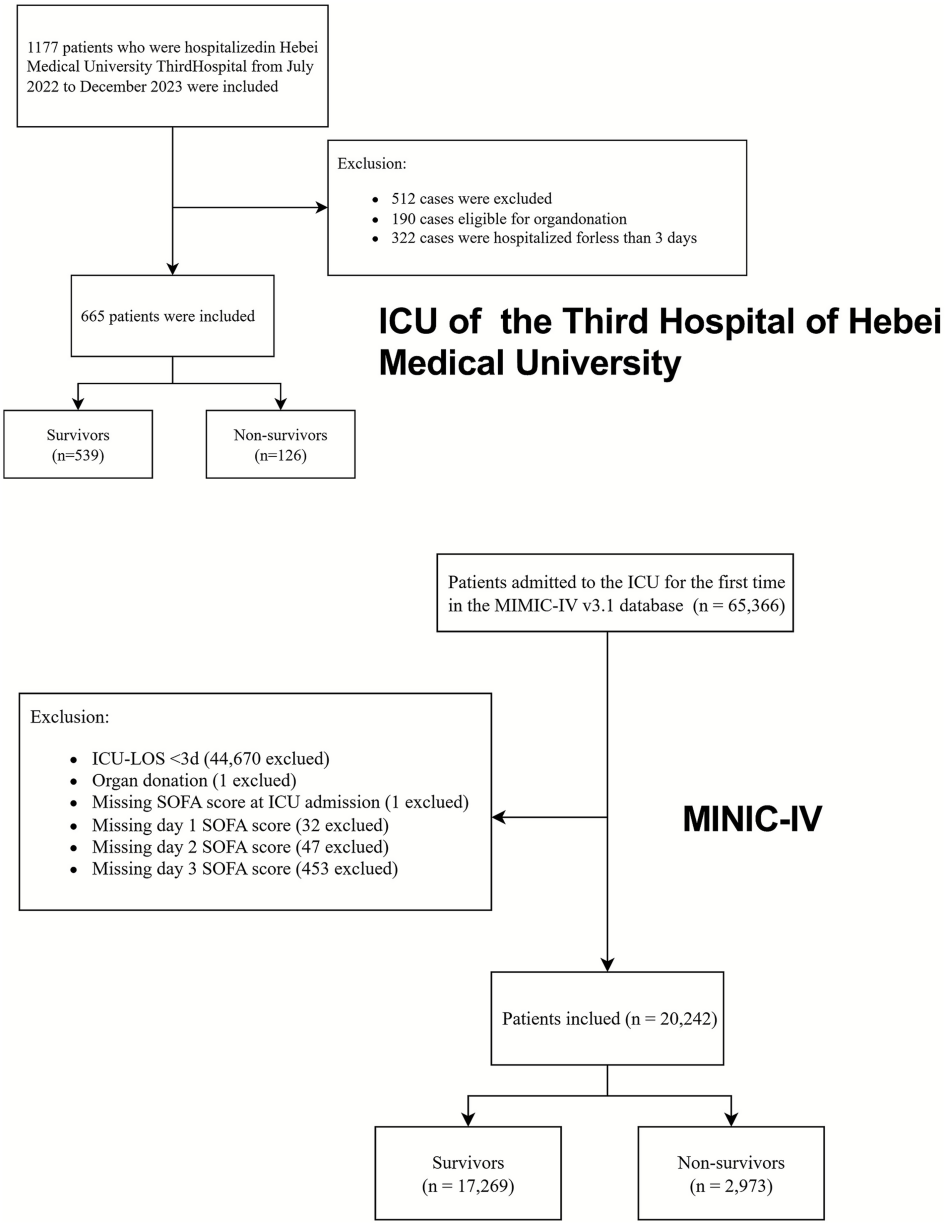
Flow chart of patients in the study cohort.

The mean age was significantly higher in the non-survivor group compared to the survivor group (64.7 ± 18.5 vs. 58.8 ± 18.1 years, *p* < 0.001). Significant differences were also observed in the APACHE II and SOFA scores between the two groups (all *p* < 0.05). Specifically, the initial SOFA score (SOFA 0) and SOFA scores recorded on days 1 (SOFA 1), 2 (SOFA 2), and 3 (SOFA 3) were significantly higher in the non-survivor group. The patient selection flowchart is shown in Figure 1, and the demographic and clinical characteristics are summarized in Table 1.

**Table 1:** Characteristics of participants.

Variable	Survivor group	Non-survivor group	*p*-value
	539 cases	126 cases	
Admission type			
Sepsis or septic shock	88 (16.3%)	46 (36.5%)	
Trauma	171 (31.7%)	24 (19.1%)	
Medical diseases	67 (12.5%)	39 (30.9%)	
Post-surgery	213 (39.5%)	17 (13.5%)	
Age (years)	58.76 ± 18.1	64.7 ± 18.5	<0.001
Gender type			0.523
Male	400 (74.21%)	90 (71.43%)	
Female	139 (25.79%)	36 (28.57%)	
APACHE II	17.23 ± 5.44	25.32 ± 7.06	<0.001
SOFA 0	6.87 ± 3.32	11.41 ± 4.28	<0.001
SOFA 1	6.54 ± 3.14	12.48 ± 4.18	<0.001
SOFA 2	6.01 ± 3.14	12.83 ± 4.61	<0.001
SOFA 3	5.63 ± 3.12	13.20 ± 4.77	<0.001

SOFA, Sequential Organ Failure Assessment; SOFA 0, SOFA score at admission to the ICU; SOFA 1, SOFA score at day 1; SOFA 2, SOFA score at day 2; SOFA 3, SOFA score at day 3; APACHE II, Acute Physiology and Chronic Health Evaluation II. A *p*-value of <0.05 was considered to be statistically significant.

**Figure 2:**
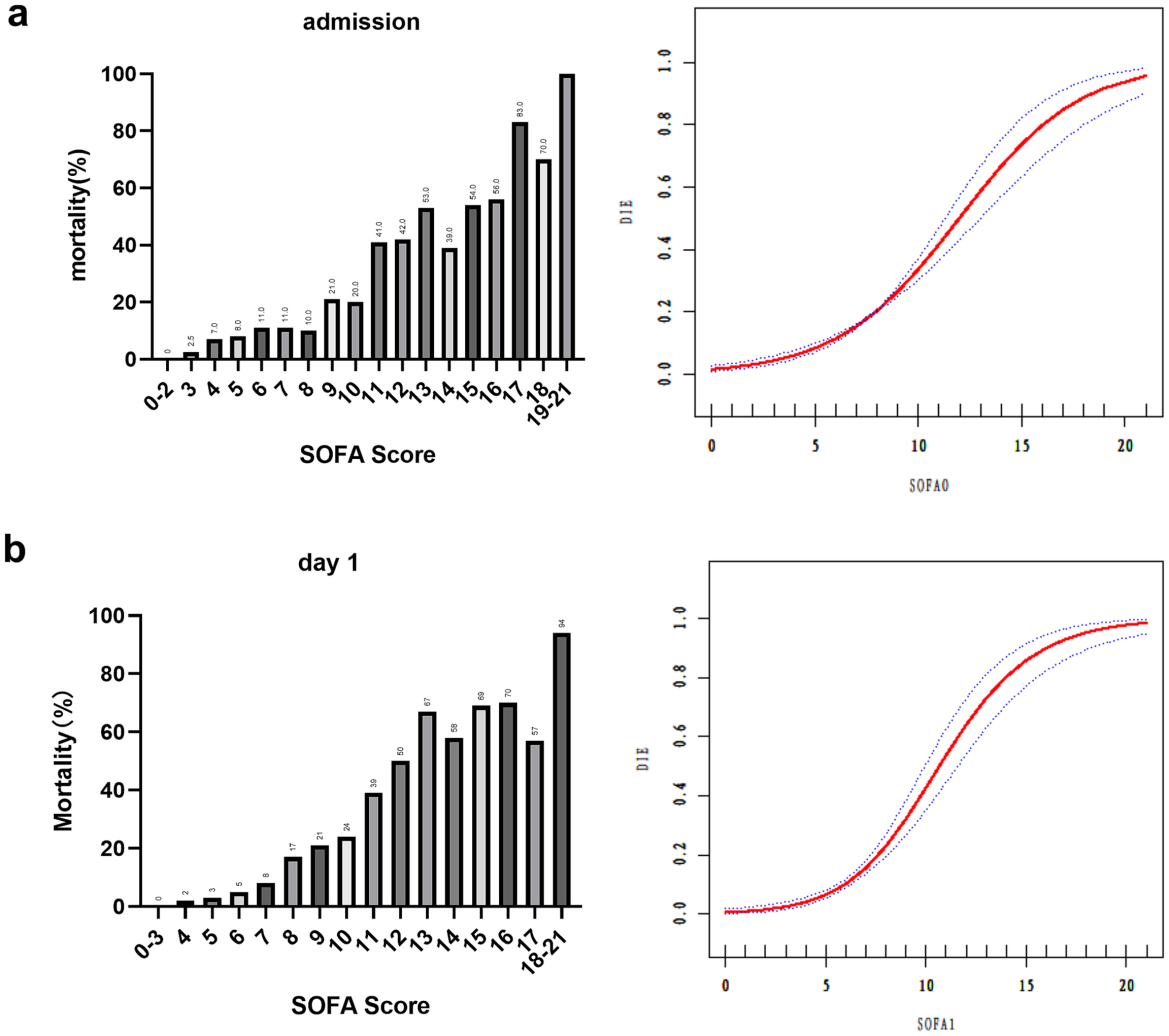
28-day mortality by admission, day 1 SOFA, and smooth curve fitting of 28-day death and SOFA. 28-day mortality and smooth curve fitting by **(a)** admission **(b)** day 1. Adjustment variable, Age; SOFA, Sequential Organ Failure Assessment; SOFA 0, SOFA score at admission to the ICU; SOFA 1, SOFA score on day 1.

As illustrated in Figure 2, the 28-day mortality rate showed a strong positive correlation with the SOFA score. Among the patients with initial SOFA scores between 0 and 3, only 1 of 85 died (1.2%), whereas all 7 patients with SOFA scores ≥18 died (100%). On day 1, all 88 patients with SOFA scores of 0–3 survived, whereas 16 of the 17 patients with scores ≥17 died (94.1%). A smoothed curve fitting analysis adjusted for age confirmed a gradual increase in mortality as SOFA scores increased.

**Figure 3:**
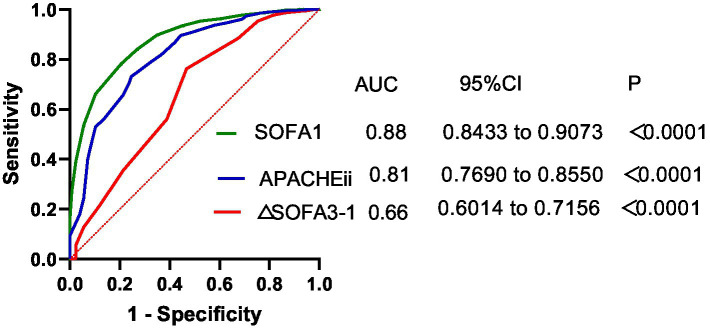
ROC curves of APACHE II, SOFA, and ΔSOFA as overall predictors of death. SOFA 1, SOFA score at day 1; ΔSOFA 3–1, SOFA score of the third day—SOFA score of the first day.

The predictive performance of the scoring systems is shown in Figure 3. The area under the ROC curve (AUC) for SOFA 1 was 0.88, outperforming the AUC for 0.81 for APACHE II. However, the AUC for ΔSOFA 3–1 was relatively lower, at 0.66, indicating limited discriminative power for mortality prediction when ΔSOFA was used alone.

**Figure 4:**
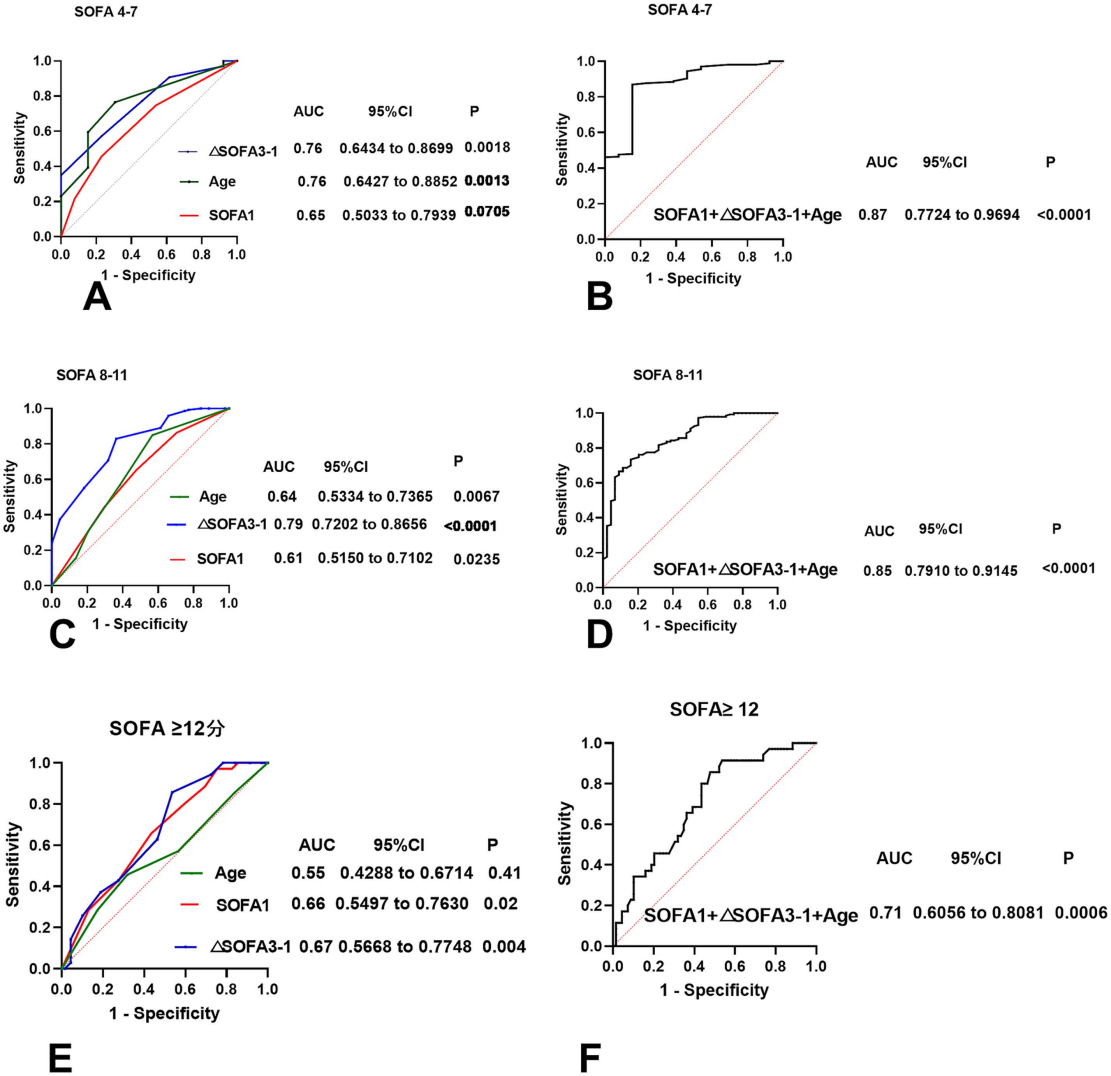
ROC curves illustrating the first-day SOFA scores across various score thresholds, along with their corresponding changes (ΔSOFA 3–1) and the combined age factor. **(A,B)** First-day SOFA scores between 4 and 7. **(C,D)** First-day SOFA scores between 8 and 11. **(E,F)** The first-day SOFA score is greater than or equal to 12.

To further explore the prognostic value, we performed a subgroup analysis stratified by SOFA 1 score ranges. ROC analyses for each subgroup are shown in Figure 4.

For SOFA 1 ≥ 12, the AUC of SOFA 1 was 0.66, whereas that of ΔSOFA 3–1 was 0.67. When combined with age (grouped by APACHE II categories), the AUC increased to 0.71.

For SOFA 1 scores between 8 and 11, the AUC of SOFA 1 was 0.61, whereas ΔSOFA 3–1 improved the AUC to 0.79. When SOFA 1 was combined with age and ΔSOFA 3–1, the AUC further increased to 0.85. The difference in AUCs was statistically significant according to DeLong’s test (*p* < 0.01), indicating improved discriminatory ability of the combined model.

For SOFA 1 scores between 4 and 7, SOFA 1 had an AUC of 0.65, and ΔSOFA 3–1 increased to 0.76. When SOFA 1 was combined with age and ΔSOFA 3–1, the AUC further increased to 0.87. The difference in AUCs was statistically significant according to DeLong’s test (*p* = 0.000976), indicating the improved discriminatory ability of the combined model.

Multivariate logistic regression analysis confirmed that the ΔSOFA score of 3–1 was an independent predictor of 28-day mortality across all SOFA 1 strata. The strongest association was observed in the 8–11 group, with an odds ratio (OR) of 73.93 (95% CI: 16.52–300.78, *p* < 0.001), as shown in Table 2.

**Table 2:** Multivariable logistic regression analyses for 28d mortality.

SOFA range		B value	OR	Multivariable	
				95%CI	*p*
8–11	SOFA 1	0.508	1.661	(1.661, 1.159)	0.006
	ΔSOFA3-1	4.303	73.927	(16.522, 300.775)	<0.001
4–7	SOFA 1	0.781	2.183	(1.156, 4.124)	0.016
	ΔSOFA 3–1	2.659	14.278	(2.942, 69.298)	0.001
≥12	SOFA 1	0.24	1.271	(1.041, 1.553)	0.019
	ΔSOFA 3–1	2.932	18.769	(2.083, 169.137)	0.009

SOFA 1: SOFA score on day 1; ΔSOFA 3–1: SOFA score of the third day—SOFA score of the first day.

**Figure 5:**
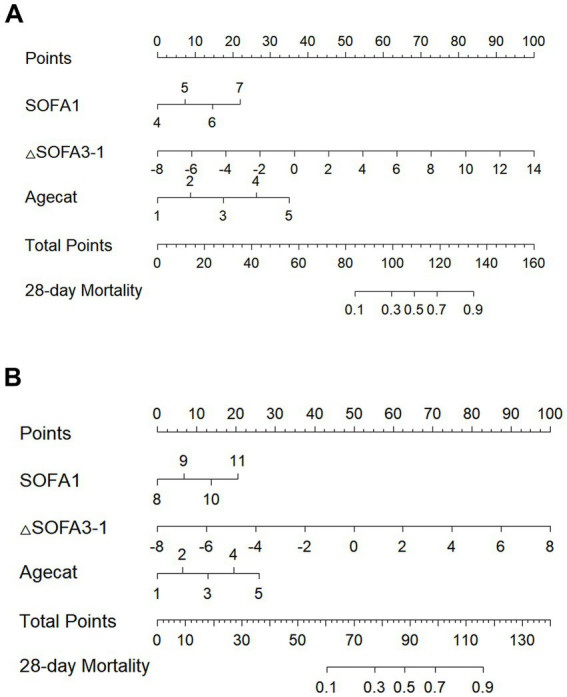
Nomograms for predicting the probability of mortality. **(A)** First-day SOFA scores between 4 and 7. **(B)** First-day SOFA scores between 8 and 11. Agecat: Categorical age variable, where participants were assigned a value of 1 for those aged 44 or younger, 2 for those aged 45–54, 3 for those aged 55–64, 4 for those aged 65–74, and 5 for those aged 75 or older.

**Figure 6:**
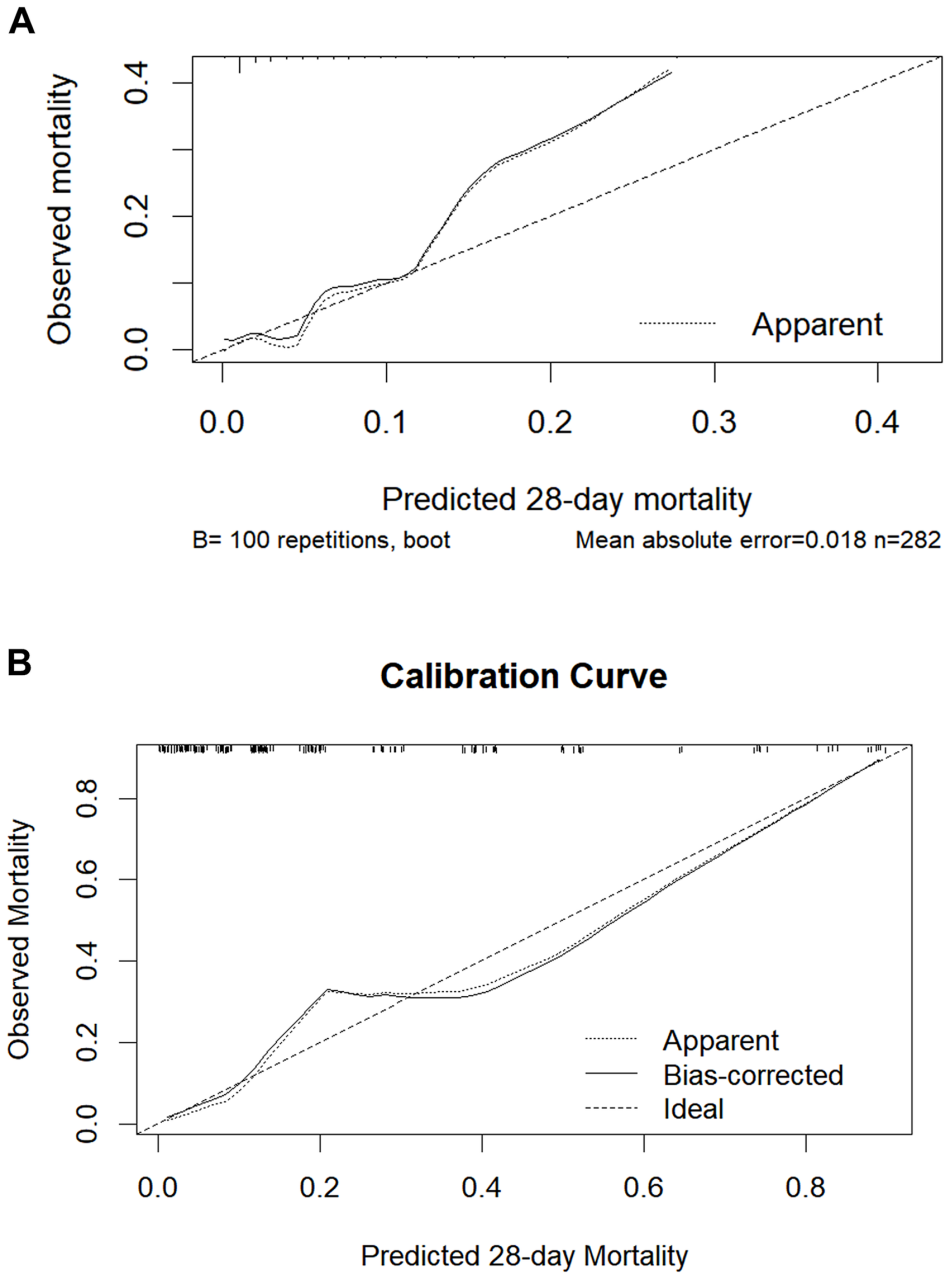
Calibration of the nomogram for mortality. The x-axis shows the predicted probability of mortality, and the y-axis shows the observed probability of mortality. **(A)** The first-day SOFA score is between 4 and 7. **(B)** The first-day SOFA score is between 8 and 11.

To facilitate the clinical application of the multivariable regression model, a nomogram integrating SOFA 1, ΔSOFA 3–1, and age was developed for SOFA 1 ranges of 4–7 and 8–11 (Figure 5) to provide an intuitive graphical representation of the predictive equation. The nomogram demonstrated excellent discriminative ability, with C-index values of 0.852 and 0.845, respectively. Calibration curves (shown in Figure 6) indicated strong agreement between the predicted and observed mortality.

**Figure 7:**
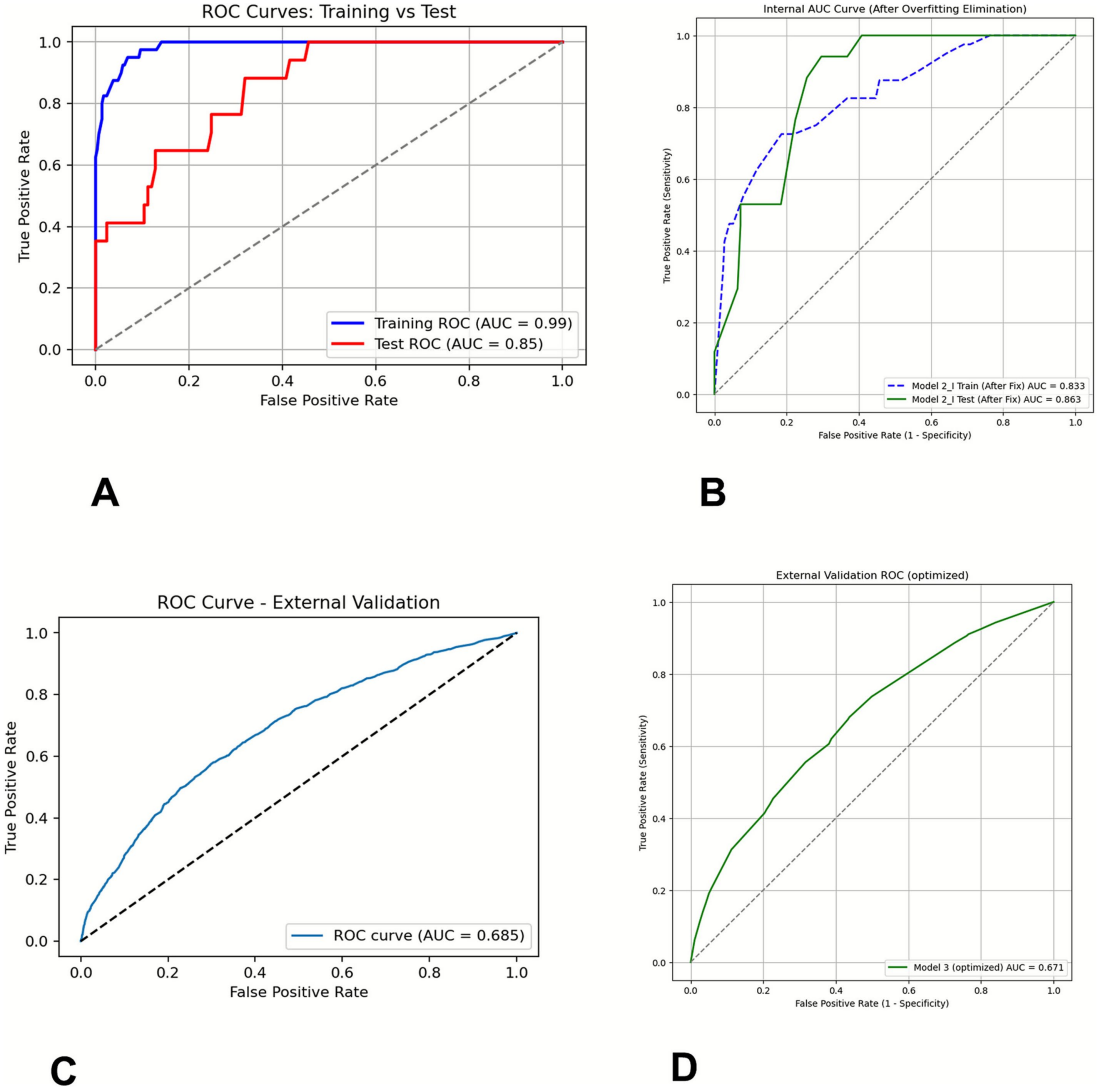
ROC curves of XGBoost models in patients with SOFA 1 scores ranging from 4 to 11 using (SOFA1, ΔSOFA 3–1, and Agecat). **(A)** Training and test sets, **(B)** training and test sets of the optimized model, **(C)** external validation set, and **(D)** external validation set of the optimized model.

To further assess the robustness and generalizability of our nomogram model, we also developed an XGBoost model using the same predictors (SOFA 1, ΔSOFA 3–1, and Agecat) in patients with SOFA 1 scores ranging from 4 to 11 (Figure 7). Comparing the performance of the machine learning model and nomogram helped evaluate the stability of the selected predictors and the potential value of incorporating non-linear relationships into risk prediction. Notably, in the internal training set, the XGBoost model achieved an AUC of 0.99 (95% CI: 0.974–0.995). In the independent internal test set, the AUC decreased to 0.85 (95% CI: 0.765–0.932).

When applied to the external validation cohort derived from the MIMIC-IV database, which included 11,207 patients with SOFA scores ranging from 4 to 11—of whom 1,122 (approximately 10.0%) died during hospitalization—the model’s AUC decreased to 0.685 (95% CI: 0.655–0.681).

To address the trade-off between model complexity and overfitting, our model was repeatedly tested and tuned, and the parameters to obtain the optimal model were determined. To ensure the stability of the model, 10-fold cross-validation was used to evaluate its predictive ability. Optimized model results (internal training AUC 0.833, internal test AUC 0.863) (Figure 7) maintained good discriminative ability. The optimized model achieved an AUC value of 0.671 in the external validation set (Figure 7).

**Figure 8:**
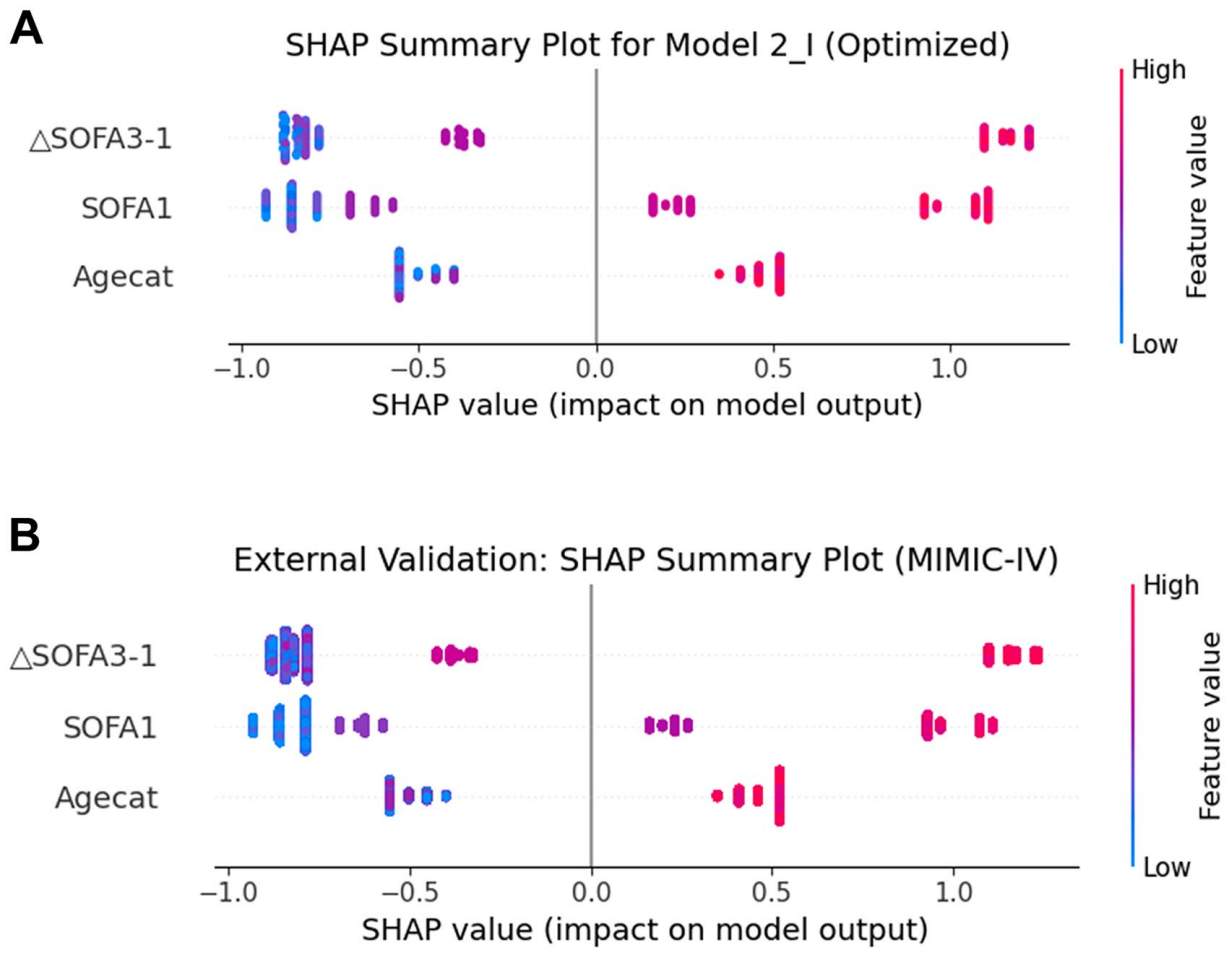
Distribution of the impacts of each feature on the XGBoost model using the SHAP value. The SHAP plot shows the dot estimation for the XGBoost model. Each dot represents the feature value of each individual patient for the model, by color (high in red, low in blue). SHAP, SHapley Additive exPlanation; XGBoost, extreme gradient boosting. **(A)** Training and test sets and **(B)** external validation set.

SHAP values were used to evaluate the potential effects of each laboratory variable on the discriminative power of the XGBoost model. The feature importance of the model is shown in Figure 8. It displays the importance of each variable on the output of the XGBoost model, from highest to lowest, according to the average absolute SHAP value. In both the internal and external datasets, SHAP analysis consistently identified ΔSOFA 3–1 (the change from day 1 to day 3) as the most influential predictor, outperforming the baseline SOFA 1 score and Agecat.

## 4 Discussion

This study highlights the prognostic value of changes in SOFA scores during ICU treatment. SOFA scores were consistently higher in non-survivors compared to survivors across all time points (admission, day 1, day 2, and day 3). The AUC of SOFA 1 was 0.8753, corroborating the findings of Raith et al. (16), who confirmed the strong prognostic performance of SOFA in predicting ICU outcomes. Notably, our study demonstrated very low mortality in patients with SOFA scores of 0–3 and sharply increased mortality with higher SOFA scores, even after adjusting for age. This pattern was consistent with prior findings by Soo et al. (4), who reported a linear association between SOFA scores and in-hospital mortality.

In our prior prospective observational study (17), we also noted a poor correlation between changes in FT3 and SOFA from day 1 to day 3, suggesting the limited prognostic reliability of ΔSOFA when considered alone. Consistently, the present study showed that ΔSOFA 3–1 had only a modest predictive value (AUC = 0.6756) for 28-day mortality in the general cohort.

Recognizing that changes in SOFA scores may have a reduced impact in patients with very high or very low initial scores, we conducted a stratified analysis by SOFA 1 ranges (4–7, 8–11, ≥12). This approach was supported by previous studies (4, 13, 18) showing a steep mortality risk beyond SOFA 11, and by others (14, 19) using 8 as a cutoff for sub-analysis. Additionally, in our cohort, all patients with SOFA scores of <4 survived, reinforcing the rationale for segmentation. Our results showed that ΔSOFA 3–1 was significantly predictive of mortality among SOFA 1 scores between 4 and 11, with the highest accuracy in the 8–11 group (AUC = 0.79). Clinically, this suggests that SOFA changes are the most informative for patients in moderate-risk categories (SOFA 1, 4, and 11), and their trends should be closely monitored during the early ICU stay.

Age is a well-established factor in determining clinical outcomes. Previous research (20) has shown that qSOFA correlates more strongly with adverse outcomes in older adults. Furthermore, combining age with SOFA scores has been demonstrated to improve prognostic accuracy (e.g., Total Max SOFA + age AUC = 0.853 vs. SOFA alone AUC = 0.845) (21). In our analysis, age was categorized based on the APACHE II age classification, and its inclusion significantly enhanced the predictive power of ΔSOFA 3–1, particularly in patients with SOFA 1 scores between 4 and 11.

Our findings suggest that integrating ΔSOFA and age into the prediction model significantly improves prognostic performance over SOFA 1 alone, as supported by DeLong’s test comparing ROC AUCs.

Nomograms offer individualized risk prediction and are increasingly used across clinical domains for disease prognosis and decision-making support (22, 23). Compared to other predictive tools, nomograms offer intuitive bedside visualization and facilitate the direct estimation of individual risk probabilities, which is especially valuable for personalized patient management. As such, the development of a nomogram helps bridge the translational gap between statistical modeling and real-world clinical decision-making. To our knowledge, no previous study has developed a nomogram incorporating ΔSOFA changes in critically ill patients. In this study, we developed a model combining SOFA 1, ΔSOFA 3–1, and age. The model demonstrated high discriminative ability, with C-index values of 0.852 (SOFA 4–7) and 0.845 (SOFA 8–11), and calibration curves indicating excellent agreement between predicted and observed mortality. These nomograms may assist ICU clinicians in identifying patients at a high risk of early death after 3 days of ICU treatment and support proactive management decisions. Nevertheless, such tools should complement, not replace, clinical judgment that accounts for patient-specific conditions such as comorbidities and physiological reserve.

To further evaluate the robustness and generalizability of our nomogram model, we developed an XGBoost machine learning model using the same predictors (SOFA 1, ΔSOFA 3–1, and Agecat) in patients with SOFA 1 scores of 4–11. Comparing the performance of the machine learning model and nomogram allowed us to assess the stability of the selected predictors and explore the potential benefits of incorporating non-linear relationships in risk prediction.

The XGBoost model demonstrated excellent performance in the internal training cohort, achieving an AUC of 0.99 (95% CI: 0.974–0.995), indicating a strong fit to the training data. However, its performance decreased to an AUC of 0.85 (95% CI: 0.765–0.932) in the independent internal test cohort, reflecting a modest decline when applied to unseen data but still maintaining good discriminative ability. Such a reduction is common in machine learning models and highlights the trade-off between model complexity and overfitting. To solve this problem, our model was repeatedly tested and tuned, and the parameters to obtain the optimal model were determined. To ensure the stability of the model, a 10-fold cross-validation was used to evaluate the predictive ability of the model. Optimized model results (internal training, AUC: 0.833; internal test, AUC: 0.863) still maintained good discriminative ability.

Notably, when applied to the external validation cohort derived from the MIMIC-IV database, the AUC of the model further decreased to 0.685 (95% CI: 0.655–0.681). The optimized model achieved an AUC of 0.671. Even after fine-tuning the XGBoost model, its predictive performance in an external validation cohort (the MIMIC-IV database) remains relatively low. We believe that this phenomenon may be attributed to several factors, including differences in data distribution, clinical practice heterogeneity, and variations in data quality across different datasets. Specifically:

### 4.1 Differences in data distribution and clinical practice

The external validation cohort was derived from the MIMIC-IV database, which includes multicenter data from various hospitals. As a result, the patient characteristics differ from those in our internal dataset. For example, age distribution, severity of illness, and clinical management approaches may vary, which could contribute to the reduced predictive ability of the model in the external validation cohort.

### 4.2 Data quality differences

In the MIMIC-IV database, SOFA scores are typically calculated using automated programs or extracted from clinical records. Although this automation improves the data collection efficiency, it may introduce inconsistencies and errors. In particular, when dealing with complex clinical data, automated scoring may not fully capture changes in a patient’s condition. Therefore, SOFA scores in the MIMIC-IV database may contain some degree of scoring errors, which could negatively impact the model’s performance on this dataset. In contrast, our internal dataset’s SOFA scores were manually corrected by clinically trained and certified physicians. Each patient’s SOFA score underwent multiple rounds of review and correction to ensure accuracy and consistency. This manual review process minimizes scoring errors to the greatest extent possible, thus enhancing the reliability of the scores.

### 4.3 Timeliness and data consistency

Additionally, because the MIMIC-IV database contains historical data, it may have certain timeliness issues. With the ongoing evolution of clinical practices and treatment strategies, data from MIMIC-IV may not fully reflect the current clinical environment. In contrast, our internal dataset is updated in real time and maintains consistency with current clinical practices.

In summary, while the MIMIC-IV database, a widely used public dataset, is highly representative, we believe that there are differences in the accuracy of SOFA scores, data quality consistency, and timeliness compared to our internal dataset. These differences may contribute to the reduced predictive performance of the model on the external dataset, especially in tasks that require highly accurate scoring. Nevertheless, the value of dynamic changes in SOFA scores remains undeniable.

To further interpret the model’s behavior, we performed a SHAP value analysis to quantify the contribution of each predictor to the model’s predictions. Notably, in both internal and external datasets, ΔSOFA 3–1 consistently emerged as the most influential predictor, surpassing the baseline SOFA 1 score and Agecat. This finding reinforces the clinical importance of dynamic SOFA score changes over time, which may capture early deterioration or improvement that static baseline scores cannot. Together, these results indicate that while the XGBoost model is capable of leveraging complex, non-linear relationships to enhance prediction, its prognostic strength is driven largely by dynamic organ dysfunction trends.

This study was conducted as a single-center, retrospective analysis, and its findings may indeed be influenced by our institution’s specific admission criteria, regional disease spectrum, and local ICU practice patterns. Therefore, caution should be exercised when extrapolating these results to other settings and healthcare systems. This retrospective study also has several inherent limitations, among which the assessment of GCS score under sedation is particularly noteworthy. A substantial proportion of ICU patients receive continuous sedation and/or analgesia, and their true level of consciousness is often obscured by medication effects. If bedside GCS values obtained during deep sedation are directly incorporated into the SOFA score, this may artificially exaggerate the degree of neurological impairment and lead to systematic overestimation of the neurological component of the SOFA score.

In future studies, we plan to further validate and extend our findings in multicenter prospective cohorts. Specifically, we aim to collaborate with ICUs from different regions and levels of care, implement harmonized SOFA scoring protocols and standardized rules for GCS assessment under sedation, and quantitatively evaluate the inter-rater reliability. We also intend to recalibrate and update the current model in larger and more heterogeneous populations and to explore decision-support tools that integrate additional dynamic indicators and electronic medical record data. Only on the basis of multicenter prospective studies can the applicability and optimal use scenarios of SOFA and SOFA-based prediction models in real-world clinical practice be more clearly defined, thereby better supporting—rather than replacing—clinical judgment.

## 5 Conclusion

In this study, we found that dynamic changes in SOFA scores during the first 3 days of ICU admission (ΔSOFA 3–1) significantly improved the prediction of 28-day mortality when combined with age and baseline SOFA 1 scores, particularly in patients with moderate illness severity (SOFA 1 scores 4–11). The developed nomogram demonstrated high discriminative ability and good calibration, offering a practical tool for individualized risk estimation at the bedside. Furthermore, the XGBoost model confirmed the robustness of the predictors and the potential value of incorporating non-linear relationships into risk prediction. However, the reduced performance in external validation emphasizes the importance of multicenter data and further validation before clinical implementation. Overall, our findings highlight the prognostic importance of early changes in organ dysfunction and support their integration into routine risk assessments in critically ill patients.

## Data Availability

The raw data supporting the conclusions of this article will be made available by the authors, without undue reservation.

## Footnotes

1
http://www.empower-stats.com/
